# Differences in E-Cadherin and Syndecan-1 Expression in Different Types of Ameloblastomas

**DOI:** 10.1155/2018/9392632

**Published:** 2018-04-23

**Authors:** Ramón G. Carreón-Burciaga, Rogelio González-González, Nelly Molina-Frechero, Sandra López-Verdín, Vanesa Pereira-Prado, Ronell Bologna-Molina

**Affiliations:** ^1^Department of Research, School of Dentistry, Juarez University of the State of Durango, Durango, DGO, Mexico; ^2^Department of Health Care, Xochimilco Unit, Autonomous Metropolitan University, Mexico City, Mexico; ^3^Research Institute of Dentistry, Health Science Center, Guadalajara University, Guadalajara, JAL, Mexico; ^4^Molecular Pathology Area, School of Dentistry, University of the Republic, Montevideo, Uruguay

## Abstract

Ameloblastomas are a group of benign, locally aggressive, recurrent tumors characterized by their slow and infiltrative growth. E-Cadherin and syndecan-1 are cell adhesion molecules related to the behavior of various tumors, including ameloblastomas. Ninety-nine ameloblastoma samples were studied; the expression of E-cadherin and syndecan-1 were evaluated by immunohistochemistry. E-Cadherin and epithelial syndecan-1 were more highly expressed in intraluminal/luminal unicystic ameloblastoma than in mural unicystic ameloblastoma and solid/multicystic ameloblastoma, whereas the stromal expression of syndecan-1 was higher in mural unicystic ameloblastoma and solid/multicystic ameloblastoma. Synchronicity was observed between E-cadherin and epithelial syndecan-1; the expression was correlated with intensity in all cases. There was a strong association between expression and tumor size and recurrence. The evaluation of the expression of E-cadherin and syndecan-1 are important for determining the potential aggressiveness of ameloblastoma variants. Future studies are required to understand how the expression of these markers is related to tumor aggressiveness.

## 1. Introduction

The ameloblastoma is an epithelial neoplasia originating in the enamel and has been described as a tumor that is usually unicentric, intermittent in growth, and persistent [1]. According to the recent classification by the World Health Organization (WHO) [2], ameloblastoma is defined as a benign epithelial odontogenic neoplasia, characterized by tumor expansion, progressive growth, and a tendency for recurrence if not completely removed. Ameloblastomas are classified as follows according to their clinical and histopathological features: solid multicystic (SMA), unicystic (UAM), extraosseous/peripheral, and metastasizing ameloblastoma; the two most common types are SMA and UAM [2]. SMA is an aggressive tumor with high recurrence rates if not treated promptly; the early manifestation of this tumor is characterized by slow growth and painless expansion, which later exhibits accelerated growth with several complications that can be fatal if its growth is not controlled. SMA has two common types of histopathological growth patterns that are not related to prognosis [2]. UAM is characterized by slow growth that occurs as a single cystic cavity, in which different types of epithelial extension can occur, namely, luminal, intraluminal (UAM-L/I), and mural (UAM-M). The mural component displays aggressive behavior, like that observed in SMA [2].

The loss of cellular adhesion plays an important role in the invasion and growth of tumor cells which are among the first events that occur in tumors of epithelial origin, such as ameloblastomas [3]. E-Cadherin (Ecad) is a calcium-dependent cell adhesion molecule that is expressed in epithelial tumors and is associated with prognosis; reduced or eliminated Ecad expression is associated with progression, invasion, and a poor prognosis in these types of tumors [4–7]. Syndecan-1 is a transmembrane proteoglycan that is expressed in fibroblasts and epithelial cells; it plays an important role in numerous biological process, such as cytoskeleton organization, cell-cell adhesion, and cell-extracellular matrix (ECM) adhesion. Syndecan-1 (Syn1) mediates interactions with ECM molecules through its heparan sulfate chains and interacts with heparin-binding growth factors, cytokines, proteinases, and proteinase inhibitors. It is considered an important structural maintenance protein [4, 8]. Syn1 is mainly localized in the basolateral surface of epithelial cells and occasionally in the stroma of mature epithelial cells [4]. The epithelial expression of Syn1 (Syn1E) has been studied in several tumors; its expression, which can range from overexpression to complete absence, has been related to tumor behavior [9–12]. The stromal expression of Syn1 (Syn1S) is related to alterations in fibronectin and ECM organization; additionally, its expression is associated with angiogenesis and it enhances the proliferation of endothelial cells and promotes the proliferation of tumor cells [13]. In ameloblastomas, the reduction or termination of expression of Syn1E is related to tumor progression and invasion, while Syn1S expression is related to cell invasion, tumor progression, and metastasis.

The expression profiles of Ecad, Syn1E, and Syn1S have been evaluated separately in previous studies [12, 14–19]. However, there is minimal research on the comparisons between the expressions of Ecad, Syn1E, and Syn1S in the same system, in which a greater understanding of the behavior of these tumors can be obtained by analyzing their expression profiles.

## 2. Materials and Methods

### 2.1. Patients

A total of ninety-nine patients treated for ameloblastoma were analyzed; clinical data and blocks of paraffin were obtained from the Oral Pathology Services of the School of Dentistry of the University of the Republic, Uruguay. Data regarding age, gender, tumor localization, tumor size, radiographical parameters, and histopathological parameters were evaluated. The histopathological diagnoses were reevaluated by two pathologists with experience in odontogenic tumors according to the latest classifications of the WHO [2]. Of the cases evaluated, there were thirty-eight cases of both UAM-L/I and SMA and twenty-three cases of UAM-M.

### 2.2. Immunohistochemistry

For immunohistochemical analyses, 3 *μ*m thick sections were prepared and mounted on slides treated with poly-L-lysine. The sections were deparaffinized in an oven at 60°C for 30 min and then incubated in xylol for 5 min. The sections were hydrated in a graded alcohol series (100, 90, 70, and 50%) and washed with distilled water. To unmask the epitopes, antigen retrieval was performed with 10 mM sodium citrate solution, with a high or low pH depending on the characteristics of each antibody. This recovery technique was performed in a microwave pressure pot with a maximum power of 750 W for two 5 min cycles, allowed to cool to room temperature, and rinsed with distilled water. Endogenous peroxidases were blocked with 0.9% hydrogen peroxide, followed by rinsing in distilled water and phosphate-buffered saline (PBS), pH 7.4. Samples were incubated for 45 min with primary antibodies against Ecad (Clone NCH-38, 1 : 100, monoclonal mouse anti-human, Dako, Santa Clara, CA, USA) and Syn1 (CD-138, Clone MIB 15, 1 : 100, monoclonal mouse anti-human, Dako). Subsequently, sections were incubated with a second biotinylated anti-mouse/anti-rabbit antibody and streptavidin/peroxidase complex (LSA-B + labeled streptavidin-biotin, Dako) for 30 min each. The products were visualized using 3,3′-diaminobenzidine-H_2_O_2_ (Dako). Fragments of epithelial tissue were used as positive controls, and incubation with the primary antibodies was omitted for negative controls.

### 2.3. Rate of Expression

Ecad and Syn1E can be observed in the cell membrane and cytoplasm of cells. The expressions of Ecad and Syn1E were calculated by light microscopy, and photomicrographs were obtained using a digital camera (Olympus C-7070) in four fields where neoplastic cells were more abundant along cystic lines and wall follicles. An average was then obtained based on the sum of the percentages of each of the fields for each case evaluated. Syn1S expression was analyzed in tumor-stromal cells and inflammatory cells, using the same technique as that for Ecad and Syn1E for the evaluation of stromal expression, without considering the expression of inflammatory cells.

### 2.4. Intensity of Expression

**Figure 1:**
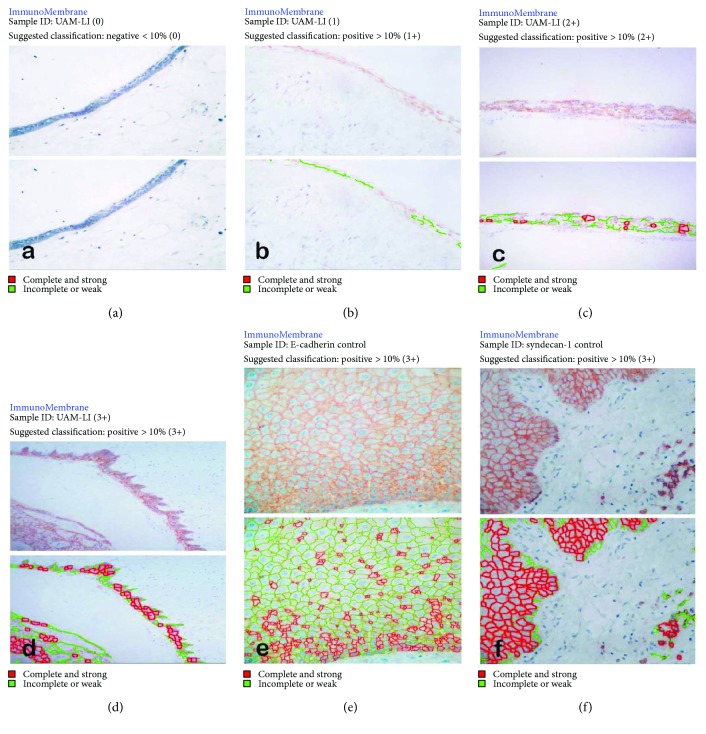
Intraluminal/luminal unicystic ameloblastomas in which the expression of E-cadherin was evaluated using ImmunoMembrane software. (a) Negative (or < 10%) immunostaining, intensity (0); (b) positive (≥10%) immunostaining, intensity (1+); (c) positive immunostaining, intensity (2+); (d) positive immunostaining, intensity (3+); (e) group control for E-cadherin expression in oral mucosa, positive immunostaining, intensity (3+); (f) group control for syndecan-1 expression in oral mucosa, positive immunostaining, intensity (3+).

In the same photomicrographs, the expression intensities of Ecad and Syn1E were also evaluated in the cell membrane using the ImageJ ImmunoMembrane plug-in (BioMediTech, Tampere, Finland). The technique was performed as reported previously by Tuominen et al. [20]. The intensity was scored as negative (0) when the intensity of membrane expression in the neoplastic cells was negative or ≤10% (Figure 1(a)), and it was scored as positive when the percentage was >10% according to whether the intensity was weakly positive (1+), moderately positive (2+), or strongly positive (3+) (Figures 1(b)–1(d), resp.). Figures 1(e) and 1(f) show the positive controls for expression of Ecad and Syn1, respectively. Owing to the stromal expression characteristics of Syn1S, its expression intensity could not be evaluated.

### 2.5. Statistical Analysis

Descriptive analysis was performed to describe the clinical features. Spearman's rank correlation coefficient (*ρ*) was used to evaluate the relationship between the rate and intensity of membrane expression. The Kruskal-Wallis test was used to determine the differences in rates of expression and clinical features. The Mann–Whitney *U* test was conducted to determine the differences in rates of expression between two groups. The results were considered statistically significant at *p* ≤ 0.05, and the rank correlation coefficient (*ρ*) was categorized as moderately positive (*ρ* = 0.50–0.70), highly positive (*ρ* = 0.70–0.90), and very highly positive (*ρ* = 0.90–1.00).

## 3. Results

### 3.1. Clinical Features and Rates of Expression

UAM-L/I and UAM-M were more frequently observed in male patients, at a ratio of 1.2 : 1 and 2.3 : 1, respectively; in contrast, the gender ratio of SMA cases was 1 : 1. With respect to age, all types were most frequently observed in patients aged < 30.4 years. Thirty-four tumors were found in the posterior region of the mandible, followed by the anterior region of the mandible with a total of three cases. The unilocular radiographic pattern was the most frequent—with a total of fifty-four cases—mostly in UAM-L/I tumors, while the multilocular pattern was more frequent in SMA tumors. A total of sixteen cases were classified as unknown cases, as they were not registered in the medical records at the time of this study. A size of <5.3 cm was the most frequent, appearing at the same frequency in UAM-L/I and SMA tumors; tumors sized >5.3 cm showed a similar distribution. Only seven cases were recurring and were identified more often in cases of SMA (Table 1).

The correlation between the expression of Ecad, Syn1E, and Syn1S and tumor size and recurrence is presented in Table 2.

### 3.2. Expression Localization

#### 3.2.1. UAM-L/I

**Figure 2:**
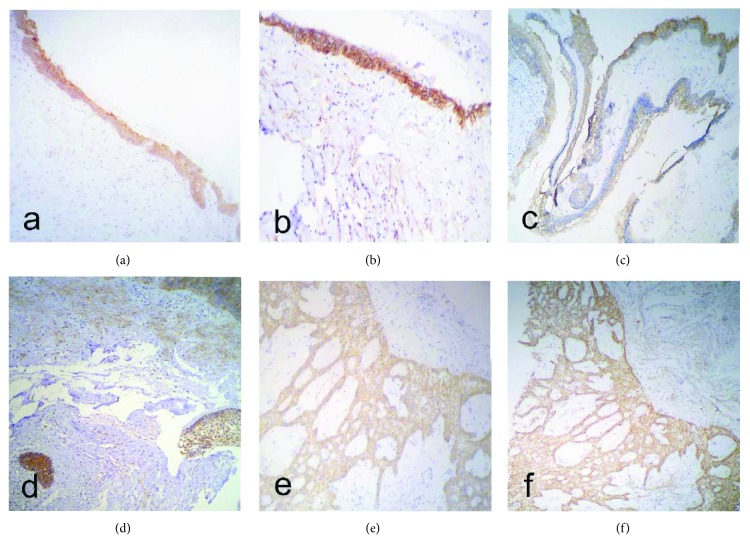
Ameloblastomas in which the rates of E-cadherin and syndecan-1 expression were evaluated. (a) E-Cadherin expression in a luminal/intraluminal ameloblastoma. (b) Expression of epithelial and stromal syndecan-1 in a luminal/intraluminal ameloblastoma. (c) Expression of E-cadherin in a unicystic ameloblastoma with mural invasion. (f) Membrane expression intensity of E-cadherin in a unicystic ameloblastoma with mural invasion. (d) Expression of epithelial and stromal syndecan-1 in a unicystic ameloblastoma with mural invasion. (e) Expression of E-cadherin in a solid/multicystic ameloblastoma. (f) Epithelial and stromal expression of syndecan-1 in a solid/multicystic ameloblastoma.

Ecad and Syn1E were expressed in the cytoplasm of tumor cells in the neoplastic cystic epithelium (luminal), in the intraluminal neoplastic cells, and in cells similar to the stellate reticulum; the highest expression was observed in neoplastic basal cells and the stellate reticulum (Figures 2(a) and 2(b)). Syn1S was present in the tumor stroma surrounding the neoplastic cells (Figure 2(b)).

#### 3.2.2. UAM-M

Ecad and Syn1E were present in the cytoplasm of the neoplastic cystic epithelium, in the intraluminal projections, and in tumor islands contained within the cystic wall. The highest rates of expression were observed in neoplastic basal cells and in the stellate reticulum (Figures 2(c) and 2(d)). Syn1S was observed in the connective tissue adjacent to both the neoplastic cystic epithelium and the neoplastic epithelial islands contained within the cystic wall (Figure 2(d)).

#### 3.2.3. SMA

Ecad and Syn1E were present in the neoplastic islands of the follicular pattern and in the cords of the neoplastic epithelium of the plexiform pattern, predominating in the tumor basal cells and in the cells resembling the stellate reticulum (Figures 2(e) and 2(f)). Syn1S was observed in the connective tissue close to the islands and cords of epithelial neoplastic cells (Figure 2(f)).

### 3.3. Expression Intensity

**Figure 3:**
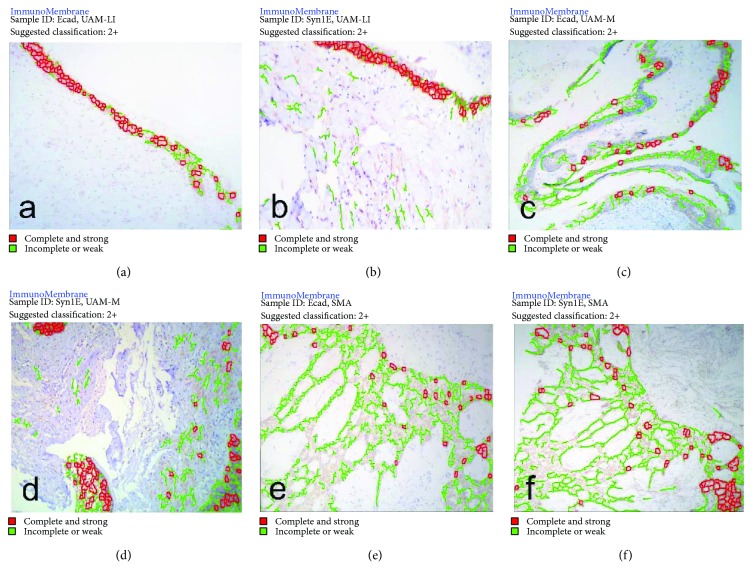
Ameloblastomas in which the membrane expression of E-cadherin and syndecan-1 were evaluated. (a) Membrane expression of E-cadherin in a luminal/intraluminal ameloblastoma. (b) Membrane intensity expression of epithelial syndecan-1 in an intraluminal/luminal ameloblastoma. (c) Membrane intensity expression of E-cadherin in a unicystic ameloblastoma with mural invasion. (d) Membrane intensity expression of epithelial syndecan-1 in a unicystic ameloblastoma with mural invasion. (e) Membrane intensity expression of E-cadherin in a solid/multicystic ameloblastoma. (f) Membrane intensity expression of epithelial syndecan-1 in a solid/multicystic ameloblastoma. The intensity of stromal expression was considered a false-positive.

Ecad and Syn1E showed similar expression levels (<1% difference); they were expressed at higher levels in UAM-L/I. Furthermore, their expression levels were significantly different only in UAM-M samples (Table 3). Syn1S expression was higher in SMA and UAM-M and differed by <5% between the two variants, while the lowest expression was observed in UAM-L/I samples (Table 3). The expression intensity of Ecad and Syn1E was 2+ in all cases, with a moderate to high positive correlation coefficient (Figure 3, Table 4).

## 4. Discussion

Ameloblastomas are locally aggressive, recurrent, benign tumors with the potential for malignant transformation into ameloblastic carcinomas [18]. UAM and SMA are most often located in the ascending limb of the mandible, followed by the body and the mandibular symphysis. Radiographically, they present as well-defined unilocular neoplasms and, in most cases, are associated with impacted dental organs, sometimes showing root resorption and cortical perforation [2]. Recent WHO guidelines [2] indicate that invasion of the cystic wall (mural invasion) by ameloblastic tumor cells in UAM is an important histopathological factor, as this mimics a behavior similar to that found in SMA [2].

In general, there are several molecular processes involved in ameloblastoma tumor progression [21]. These include proteins related to cell adhesion, apoptosis regulation, cell cycle, cell proliferation, BRAF V600 mutations, tumor front, angiogenesis, bone remodeling, and the ECM [22–29].

Cell adhesion molecules are altered in various neoplasms, especially malignant ones, favoring progression, invasion, recurrence, and metastasis [3, 30]. Ecad is a molecule with tumor suppressor functions; it is possible that these functions are related to the capacity of cells to adhere because in normal cells, growth and migration are inhibited when one cell adheres to another [30]. The loss of Ecad in most tumors of epithelial origin may be associated with aggressiveness, tumor invasion, and metastasis [4, 30, 31]. Our results show that the rate of Ecad expression was the highest in UAM-L/I tumors. Ecad expression was highly similar between UAM-L/I and UAM-M samples, suggesting that Ecad expression may be related to a decrease in cell adhesion capacity and, therefore, to the potential for the tumor cell to invade the cystic wall. This possibility could be supported by the results obtained for SMA, whose expression profiles were lower than those for UAM-L/I and UAM-M.

Martinez-Martinez et al. [19] reported that the expression levels of Ecad and B-catenin were low in ameloblastomas and ameloblastic carcinomas, consistent with our results. This suggests that Ecad is associated with tumor behavior due to the loss of cell adhesion. Ecad expression in cells resembling the stellate reticulum was observed in the three tumor variants included in this study, which could demonstrate, as shown by Pereira et al. [17], that increased expression of Ecad promotes adhesion between distant cells, as observed in UAM-L/I tumors.

Syndecans are a family of heparan sulfate proteoglycans that are expressed on the surface of adherent cells and in many nonadherent cells. Syndecans consist of a family of four members; Syn1 is the most important and most well-characterized. Syn1 plays a major role in cell-cell and ECM adhesion, interacts with various heparin-binding growth factors, and is an important regulator of cell adhesion and ECM molecules [16, 24, 32]. In tumor biology, Syn1 has been reported as a prognostic indicator for several malignancies, as its expression levels have a strong association with tumor invasion, proliferation, and cellular adhesion [12]. In head and neck carcinomas, decreased or absent Syn1 expression is associated with aggressiveness and a poor prognosis [14, 25].

The results of our study showed that the rate of Syn1E expression was higher in UAM-L/I, consistent with those of previous studies [12]. Interestingly, Ecad and Syn1E had a difference in expression of <1%; therefore, as in previous studies [26], it is possible that the minimal difference in Ecad and Syn1E expression in ameloblastomas is related to the coordination and regulation of expression between these two proteins and that the synchronous increase or decrease in expressed is associated with the adhesive capacity of tumor cells. Several studies have associated the synchronous reduction of Ecad and Syn1E with the processes of carcinogenesis and used it as an indicator of prognosis [26]. Syn1S expression is related to the regulation of cell growth, cell proliferation, the capacity to interact with heparin-binding growth factor family members, induction of angiogenesis, growth promotion, and tumor progression; it contributes to the invasion and metastasis of malignant tumors [14, 25].

Our data suggest that UAM-L/I tumors have cohesive cell groups that possess a lower invasive capacity and result in a better prognosis, unlike UAM-M tumors, which display less cohesive cell groups that possess a more invasive phenotype, resulting in bone reabsorption, cystic growth, and subsequent mural invasion. SMA tumors are more aggressive than UAM tumors [2]; our results show that UAM-L/I exhibited a higher expression of Ecad and Syn1E, but not Syn1S, which was expressed at lower levels than in SMA. It is interesting to note that UAM-M tumors were found to be highly similar to SMA. In our study, a low difference in Syn1S expression was observed between UAM-M and SMA tumors, which could indicate that the observed expression patterns are related to the similar behaviors of the UAM-M and SMA tumors; conversely, UAM-L/I tumors display less aggressive behavior, consistent with the recent WHO guidelines, which noted that UAM with mural invasion displays behaviors similar to SMA [2].

We did not find any associations between clinical characteristics such as gender, age, localization, and radiographical patterns; however, we found that the expression of Syn1S was somewhat higher in males, patients aged < 30.4 years, the posterior region of the mandible, and multilocular radiographic patterns, suggesting that these data may be associated with tumor aggressiveness. The radiographic characteristics seem to be relevant for the prognosis of ameloblastoma, as the multilocular radiographic pattern was found to be related to a higher incidence of recurrence [33]. In our study, the multilocular radiographic pattern was more frequently associated with SMA, and this variant was more frequently associated with recurrence. Therefore, the multilocular radiographic pattern is an important risk factor that should be considered at the time of surgical treatment, as it is possible that the multilocular pattern is associated with cell invasion.

We found that the expression of Ecad and Syn1E in recurrent ameloblastomas was minimal compared to that in nonrecurrent ameloblastomas; although the difference in expression was not high, it was significant, especially between Syn1E and Syn1S. This result could indicate that tumor invasion is related to alterations in cell-cell and cell-ECM interactions, mediated by cell adhesion molecules [34]; it is possible that the gradual loss of the expression of Ecad and Syn1E as well as the gradual increase in Syn1S expression are related to the promotion of tumor cell invasion to the stroma, and with the capacity to gain migratory capability and autonomous cell survival. This hypothesis is supported by our results, which showed a higher expression of Ecad, Syn1E, and Syn1S in ameloblastomas ≥ 5.3 cm in size compared to those < 5.3 cm in size. This could indicate that the growth of ameloblastomas is slow and constant and that during growth, these cells acquire a migratory and invasive capacity, forming tumor nests related to recurrence.

## 5. Conclusion

In this study, we evaluated clinical, radiographic, and histopathological findings from ninety-nine cases of ameloblastoma and related these data to the expression of Ecad, Syn1E, and Syn1S. Importantly, we found that the expression of these proteins had an association with radiographic patterns and tumor size as important risk factors for recurrence. We established a possible relationship between Ecad and Syn1E expression in ameloblastomas; a synchronous reduction in Ecad and Syn1E expression was found to be associated with a high expression of Syn1S. This is a potential factor contributing to the aggressive behavior of these tumors. Additional studies with more ameloblastoma cases are required and should include clinical follow-up and coexpression studies in which Ecad and Syn1 are evaluated with proteins involved in cell proliferation, apoptosis, and growth factor proteins, as well as proteins involved in bone remodeling and ECM interactions. This can help to determine whether Syn1S expression and the simultaneous decreases in Ecad and Syn1E expression are also associated with mechanisms of bone remodeling, tumor invasion, and cellular immortality.

## Figures and Tables

**Table 1:** Distribution of ameloblastomas according to clinical features.

Clinical features	UAM-L/I (*n* = 38)	UAM-M (*n* = 23)	SMA (*n* = 38)
Gender			
Male	21 (55.3%)	16 (69.6%)	19 (50%)
Female	17 (44.7%)	7 (30.4%)	19 (50%)
Mean age, years			
<30.4	26 (68.4%)	14 (60.9%)	17 (44.7%)
≥30.4	12 (31.6%)	9 (39.1%)	21 (55.3%)
Site of localization			
Maxilla			
Anterior	2 (5.3%)	0	0
Posterior	1 (2.6%)	0	0
Mandible			
Anterior	1 (2.6%)	1 (4.3%)	1 (2.6%)
^∗^Posterior	34 (89.4%)	22 (95.7%)	37 (97.4%)
Radiographic			
Unilocular	26 (68.4%)	16 (69.6%)	12 (31.6%)
Multilocular	8 (21%)	3 (13%)	18 (47.4%)
Unknown	4 (10.5%)	4 (17.4%)	8 (21%)
Size (cm)			
<5.3	23 (60.5%)	17 (74%)	23 (60.5%)
≥5.3	15 (39.4%)	6 (26%)	15 (39.5%)
Recurrence			
Yes	2 (5.3%)	2 (8.7%)	3 (7.9%)
No	36 (94.7%)	21 (91.3%)	35 (92.1%)

$\begin{array}{c} {}^{*} \end{array}$Body and/or ramus.

**Table 2:** Percentages of E-cadherin and syndecan-1 expression and their relationship with the clinical parameters.

Expression, versus	Gender (SD)	Mean age, years (SD)	Maxilla and mandible (SD)	Radiographic pattern (SD)	Tumoral size, cm (SD)	Recurrence (SD)
	Male/female	<30.4/≥30.4	Anterior/posterior	Unilocular/multilocular/unknown	<5.3/≥5.3	Yes/no
% Ecad	61.1 ± 26.7/52.7 ± 32.6	60.4 ± 30.1/53.4 ± 28.8	Maxilla: 85± 7.1/60 Mandible: 70± 10/56.4 ± 30.1	57.6 ± 32/54.5 ± 27.3/62.8 ± 23.9	55.2 ± 30.4/61.3 ± 28	57.1 ± 26.2/57.5 ± 29.9
% Syn1E	59.6 ± 24.9/54.7 ± 26.9	56.5 ± 26.7/58.9 ± 24.8	Maxilla: 55± 21.2/70 Mandible: 63.3 ± 20.8/57.3 ± 26	57.5 ± 26.2/58.9 ± 25.3/55± 26.1	55± 26.6/62± 24.1	47.1 ± 33/58.3 ± 25.2
% Syn1S	47.8 ± 27.8/43.9 ± 28.1	40.3 ± 28.3/54± 25.6	Maxilla: 20± 28.3/30 Mandible: 41.7 ± 36.2/46.9 ± 27.8	41.3 ± 26.7/52.9 ± 27.7/48.4 ± 29.9	46± 28.1/46.2 ± 27.9	44.3 ± 32.6/46.2 ± 27.7
^∗^ *p* =	0.651	0.424	0.690	0.104	*0.049*	*0.027*

$\begin{array}{c} {}^{*} \end{array}$Kurskal-Wallis test, significance at *p* < 0.05. Statistical significance was found between the percentage expression and tumoral size and recurrence. Ecad = E-cadherin expression; Syn1E = epithelial expression of syndecan-1; Syn1S = stromal expression of Syndecan-1.

**Table 3:** Differences in expression among UAM-L/I, UAM-M, SMA, and E-cadherin and syndecan-1 expression.

Ameloblastoma	^∗^ *p* values		
	% Ecad versus % Syn1E	% Ecad versus % Syn1S	% Syn1E versus % Syn1S
UAM-L/I	0.088	*0.001*	*0.001*
UAM-M	*0.010*	*0.012*	*0.010*
SMA	0.881	0.180	0.123

$\begin{array}{c} {}^{*} \end{array}$Mann–Whitney *U* test, statistical significance at *p* < 0.05. Ecad = E-cadherin expression; Syn1E = epithelial expression of syndecan-1; Syn1S = stromal expression of syndecan-1.

**Table 4:** Correlation between percentage expression and intensity of E-cadherin and syndecan-1 in UAM-L/I, UAM-M, and SMA.

Ameloblastoma	% Ecad (SD)/intensity	Spearman (*ρ*)	% Syn1E (SD)/intensity	Spearman (*ρ*)	% Syn1S (SD)
UAM-L/I	65.5±32.6/2	*ρ* = 0.830	64.7 ± 24.8/2	*ρ* = 0.777	36.4±24.9
UAM-M	58±23.6/2	*ρ* = 0.655	58.9 ± 22.1/2	*ρ* = 0.744	49.1±19
SMA	49±28/2	*ρ* = 0.573	49.5 ± 27.1/2	*ρ* = 0.838	53.9±32.6
^∗^Significance		*p* = 0.001		*p* = 0.001	

^∗^Statistical significance at *p* < 0.05, expression versus intensity. Ecad = E-cadherin expression; Syn1E = epithelial expression of syndecan-1; Syn1S = stromal expression of syndecan-1.
